# Evaluating the Accuracy and Completeness of Artificial Intelligence Responses Against Established Otology Guidelines

**DOI:** 10.1097/ONO.0000000000000059

**Published:** 2024-08-12

**Authors:** Nicholas A. Rossi, Kassandra K. Corona, Yuki Yoshiyasu, Dayton L. Young, Brian J. McKinnon

**Affiliations:** 1 Department of Otolaryngology, University of Texas Medical Branch, Galveston, Texas; 2 School of Medicine, University of Texas Medical Branch, Galveston, Texas.

**Keywords:** Artificial intelligence, Hearing loss, sudden, Otitis media, Otology, Tinnitus

## Abstract

**Background:**

The incorporation of artificial intelligence (AI), especially large language models like Generative Pretrained Transformer 4 (GPT-4), into medical practice is a burgeoning field of interest. This research evaluates the applicability of GPT-4 in otology by analyzing its responses to queries based on otologic clinical practice guidelines.

**Methods:**

Key guidelines from otology were selected, and corresponding questions were formulated to examine GPT-4’s interpretation and response accuracy. Two independent reviewers assessed the AI-generated answers for accuracy and completeness, using a structured Likert scale. A re-evaluation was conducted to evaluate the reproducibility of the results.

**Results:**

The analysis showed a high accuracy level (mean score: 4.75 of 5) and completeness (mean score: 2.88 of 3) in GPT-4’s responses. The interrater agreement, as indicated by Cohen κ, was substantial. GPT-4 consistently advised on individualized treatment plans and professional consultation, particularly for guidelines with weaker evidence, demonstrating its cautious approach to handling medical information.

**Conclusion:**

GPT-4 exhibits promising potential as an auxiliary tool in otology, providing accurate and comprehensive information. However, its role should be viewed as supplementary, with emphasis on continual updates and careful monitoring to align with evolving medical knowledge. Future studies are recommended to further explore the depth of AI application in diverse clinical scenarios and its real-time impact on clinical outcomes.

The advent of large language models has marked a significant milestone in the field of artificial intelligence (AI). These models utilize deep learning algorithms, trained on extensive internet sources, to predict word sequences and generate text that closely resembles human language. One of the most advanced among these, the Generative Pretrained Transformer 4 (GPT-4), developed by OpenAI, has rapidly gained recognition for its sophisticated capabilities (1–4). GPT-4 has shown remarkable potential, notably its ability to pass the US Medical Licensing Examination Step exams as well as outperforming medical students and residents on board-style questions (5,6), highlighting its potential utility in medical education and information processing.

GPT-4, developed by OpenAI, represents a significant advancement in AI through its capability to generate text that closely mimics human language based on the input it receives. It is trained on a vast array of data sources, including texts from the public domain, websites, and other forms of publicly available information, up to a specific cutoff date prior to its release. This extensive training allows GPT-4 to draw upon a wide range of information to generate responses. However, the specific datasets used and the weighting given to various sources in its training algorithm remain proprietary details held by OpenAI. Although GPT-4 does not permit custom training by end-users on specific datasets, its performance can be influenced by the manner in which queries are structured and the information available to it up to its last training update. This background is crucial for understanding the capabilities and limitations of using GPT-4 in medical and other specialized fields of inquiry.

The application of AI language models like GPT-4 has not yet been comprehensively explored in the realm of otology. One major challenge is ensuring the accuracy and reliability of the information generated by these models, as they are based on algorithms that may not always align with the latest or most specific clinical guidelines. This study aims to methodologically evaluate the potential of AI in interpreting otological guidelines, thereby contributing to the development of research techniques in this field. In otology, several key clinical practice guidelines (CPGs) published by the American Academy of Otolaryngology—Head and Neck Surgery Foundation (AAO-HNSF) serve as the cornerstone for clinical decision-making, such as those pertaining to otitis media, sudden hearing loss, and Meniere disease (7–14). These guidelines are periodically updated to reflect the latest evidence and expert consensus. The methodological objective of this study is to evaluate the accuracy and comprehensiveness of responses generated by GPT-4 in relation to established otology guidelines. By comparing AI responses to the current standards set forth in otology CPGs, this study aims to assess the potential role and limitations of AI language models in supporting otologic medical practice and education.

## MATERIALS AND METHODS

This study focused on GPT-4’s analysis of the 8 otology-specific CPGs published by the AAO-HNSF. These CPGs are listed in Table 1.

**TABLE 1.** Key otology clinical practice guidelines analyzed in the study

No.	Authors	Year	Title of clinical practice guideline
1	Basura GJ, Adams ME, Monfared A, et al.	2020	Clinical practice guideline: Ménière’s disease
2	Baugh RF, Basura GJ, Ishii LE, et al.	2013	Clinical practice guideline: Bell’s palsy
3	Bhattacharyya N, Gubbels SP, Schwartz SR, et al.	2017	Clinical practice guideline: benign paroxysmal positional vertigo (update)
4	Chandrasekhar SS, Tsai Do BS, Schwartz SR, et al.	2019	Clinical practice guideline: sudden hearing loss (update)
5	Rosenfeld RM, Shin JJ, Schwartz SR, et al.	2016	Clinical practice guideline: otitis media with effusion (update)
6	Schwartz SR, Magit AE, Rosenfeld RM, et al.	2017	Clinical practice guideline (update): earwax (cerumen impaction)
7	Tunkel DE, Bauer CA, Sun GH, et al.	2014	Clinical practice guideline: tinnitus
8	Rosenfeld RM, Schwartz SR, Cannon CR, et al.	2014	Clinical practice guideline: acute otitis externa

Key action statements, along with their respective grade of evidence and level of recommendation, were extracted from the clinical practice guideline. For each key action statement, corresponding questions were carefully formulated to encapsulate the essence of the guideline (Supplemental Content, http://links.lww.com/ONO/A31). These questions were designed to probe the depth and breadth of GPT-4’s understanding and interpretation of otology practices.

This study involved the analysis of publicly available clinical guidelines and AI-generated responses without involving human or animal subjects. Accordingly, it was deemed exempt from Institutional Review Board (IRB) approval per the standing IRB guidelines at the University of Texas Medical Branch.

### AI Response Generation and Evaluation

The formulated questions were input into GPT-4 on October 21, 2023. Two study authors were tasked with evaluating the AI-generated responses. The assessment criteria were 2-fold: accuracy and completeness. Accuracy was graded on a Likert scale ranging from 1 to 5 (Table 2). Completeness was graded similarly on a Likert scale from 1 to 3 (Table 3).

**TABLE 2.** Likert scale for grading accuracy of AI responses in otology guidelines

1	Completely incorrect
2	More incorrect than correct
3	Approximately equal correct and incorrect portions
4	More correct than incorrect
5	Completely correct

AI indicates artificial intelligence.

**TABLE 3.** Likert scale for grading completeness of AI responses in otology guidelines

1	Incomplete, addressing some but not all critical aspects of the question
2	Adequate, addressing all aspects of the question
3	Comprehensive, addressing all aspects and providing additional relevant information

AI indicates artificial intelligence.

In determining the scales for assessing the accuracy and completeness of AI responses, we chose a 1–5 Likert scale for accuracy to capture the nuanced differences reflective of detailed medical information. Conversely, for completeness, a 1–3 scale was selected, considering the relatively straightforward nature of evaluating whether the response adequately covered the guideline’s key points. This approach was intended to balance the need for detailed assessment of accuracy with the simplicity of evaluating completeness.

To ensure the validity of our study methodology, it is crucial to address potential concerns regarding the alignment of the publication dates of CPGs with the information available to GPT-4. At the outset of our investigation on October 21, 2023, the data input to GPT-4 reflects the most current information available up to that date. While the specific timeline for data inclusion in GPT-4 is proprietary and not disclosed, our study design aimed to capture responses reflective of the latest medical knowledge accessible to the model at the time of our inquiry. We recognize the importance of ongoing updates to AI models like GPT-4 to maintain their relevance in the context of evolving medical guidelines.

### Reproducibility and Statistical Analysis

To assess the reproducibility of results and GPT-4’s adaptability over time, the AI was requeried on November 23, 2023, using the same questions to confirm reproducibility of results. These updated responses were then reassessed by the same reviewers. The statistical analysis of the data, including the calculation of descriptive statistics and interrater reliability, was conducted using Microsoft Excel (2016 version, Microsoft Corporation, Seattle, WA). Interrater reliability was quantified using weighted Cohen κ. This statistic accounts for the agreement occurring by chance, providing a more robust measure of consistency between raters.

Considering the dynamic nature of AI model training and refinement processes, the selection of query dates and the rationale for assessing reproducibility within a relatively short timeframe require clarification. GPT-4 undergoes periodic updates and refinements to enhance its performance and incorporate new data. While the specific frequency of these updates is proprietary, our study aimed to evaluate the consistency of GPT-4’s responses over a brief interval. The decision to requery GPT-4 within a month was intended to assess its stability and adaptability, ensuring a robust evaluation of its capabilities.

### Verification of CPG Accuracy

To address concerns regarding the potential outdated nature of some CPGs used in our study, 2 board-certified neurotologists reviewed the identified errors attributed to ChatGPT to confirm whether these errors were due to ChatGPT’s inaccuracies or the possibility of outdated information in the CPGs. This verification process was carried out for each guideline where discrepancies were noted, ensuring the validity of our findings.

## RESULTS

**FIG. 1.**
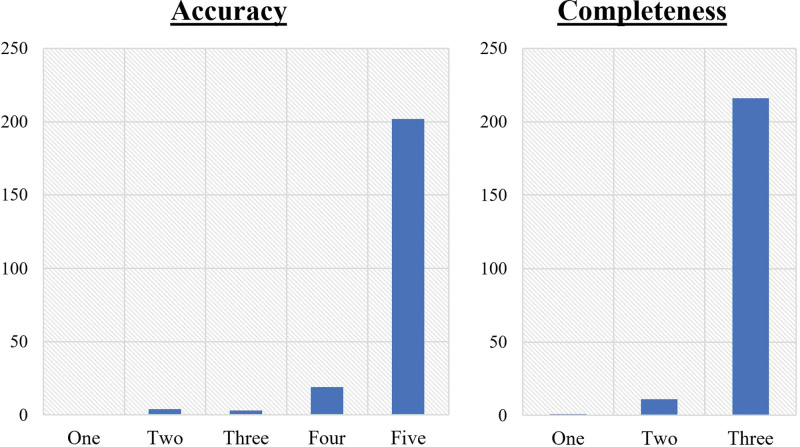
Graphical representation displaying the distribution of scores on the Likert scale for accuracy and completeness.

Overall accuracy and completeness scores were determined to be 4.75 and 2.88, respectively. The distribution of scores for both accuracy and completeness is detailed in Table 4 and Figure 1. The interrater agreement between the 2 reviewers, as measured by weighted Cohen κ, was calculated to be 91.1% (Cohen κ coefficient = 0.74), indicating a substantial agreement in scoring.

**TABLE 4.** Comparative analysis of accuracy and completeness scores

	Mean accuracy score	SD	Mean completeness score	SD
Author 1	4.74	0.56	2.88	0.32
Author 2	4.76	0.24	2.89	0.31
Combined	4.75	0.44	2.88	0.32

All AI responses in this study included a qualifier emphasizing the importance of individualized treatment plans and consultation with healthcare professionals. This pattern reflects an inherent aspect of AI responses in the medical domain, acknowledging the personalized nature of healthcare.

There were a few notable instances where GPT-4 responses deviated from the expected accuracy. For example, when queried regarding the recommendation of postural restrictions following canalith repositioning procedures (CRPs), GPT-4 stated: “In summary, the use of postprocedural postural restrictions after CRP for posterior canal BPPV is a matter of clinical judgment.” GPT-4 cited varying evidence regarding postural restrictions post-CRP, despite most high-level data and meta-analyses revealing no effect on outcomes (9,15). Furthermore, GPT-4 suggested the prophylactic use of intratympanic steroids in Meniere disease as “showing considerable promise,” a statement not supported by current guidelines, and overgeneralized the effectiveness of diuretics and betahistine in treating Meniere disease, implying broad efficacy “for most patients.” When these questions were requeried, the regenerated responses remained largely unchanged, confirming the initial inconsistencies. These inaccuracies highlight the need for critical evaluation of AI-generated medical advice against up-to-date clinical guidelines.

### Reproducibility Analysis

The reproducibility of GPT-4’s responses was evaluated by requerying the model with the same set of questions on November 23, 2023, following the initial query on October 21, 2023. The mean accuracy and completeness scores for the re-evaluated responses were found to be consistent with those obtained during the initial assessment. Specifically, the accuracy score remained at 4.75 of 5, while the completeness score remained at 2.88 of 3. These findings indicate a high level of stability in GPT-4’s performance over the 1-month interval.

### Verification Outcomes

It was confirmed that in all cases, the inaccuracies or incomplete statements were attributable to ChatGPT and not due to outdated CPGs. This ensured that the discrepancies highlighted in our study were genuinely reflective of ChatGPT’s limitations rather than temporal misalignments in guideline updates.

## DISCUSSION

The integration of AI technology such as GPT-4 into medical practice, especially in specialized fields like otology, is an area of burgeoning interest but requires careful consideration and extensive validation (1–4). This study highlights GPT-4’s potential in accurately interpreting and responding to otology-related queries, demonstrating a high degree of accuracy (mean score of 4.75 of 5) and completeness (mean score of 2.88 of 3) in its responses to questions about diagnosis, pathophysiology, management, and complications of ear-related diseases.

GPT-4’s knowledge base is restricted to information available up to a certain date, which in this case included the latest updates in otology guidelines. This temporal limitation might partly explain the high accuracy observed in our study. As medical knowledge and guidelines evolve, it is crucial for AI models to be continually updated to maintain their relevance and accuracy. In our analysis, GPT-4 demonstrated a cautious approach, often qualifying its responses with reminders of its limitations as an AI tool and advising consultation with healthcare professionals. This characteristic could be beneficial in reminding users of the AI’s auxiliary role in medical decision-making.

In the broader context of AI applications in otolaryngology, the findings of this study align with those of Yoshiyasu et al, who evaluated GPT-4’s performance against the “International Consensus Statement on Allergy and Rhinology: Rhinosinusitis in 2021” (4). Yoshiyasu et al reported high accuracy (mean score of 4.26 of 5) and substantial completeness (mean score of 2.72 of 3) in GPT-4’s responses, with strong interrater agreement, similar to the findings of the current study on otology guidelines. This underscores the consistent capabilities of GPT-4 across different otolaryngologic subspecialties in interpreting and responding to guideline-based queries. Both studies highlight the model’s tendency to provide qualified responses and to identify areas of less definitive guidance. However, they also emphasize the necessity of continual updates to the AI model and its role as a supplemental tool to physicians.

While this study underscores the potential of GPT-4 in otology, it is important to acknowledge its limitations. The selection of questions, albeit designed to reflect key aspects of otologic CPGs, was inherently subjective. This approach may not encompass the full spectrum of clinical scenarios and patient queries encountered in real-world practice. The AI’s responses were based on a predefined dataset, which may not include the latest updates or diverse perspectives in the rapidly evolving field of otology. Consequently, the study’s findings might not fully represent GPT-4’s capability to process emergent medical information or nuanced clinical cases. Additionally, the study focused solely on guidelines from the AAO-HNSF, potentially excluding other influential sources and international perspectives in otology. The impact of AI assistance on actual patient outcomes, clinical decision-making processes, and workflow efficiency was beyond the scope of this study.

The potential for integrating AI tools like GPT-4 into primary care settings presents a promising avenue for enhancing adherence to CPGs, particularly in areas where adherence has historically been low, such as the treatment of otitis externa. By providing immediate, evidence-based recommendations, AI can support primary care physicians in making informed decisions that align with the latest guidelines. This not only improves the quality of care delivered to patients but also bridges knowledge gaps that may exist due to the vast and ever-growing volume of medical literature. Furthermore, the cautious nature of AI-generated responses, with explicit reminders of the importance of professional judgment and individualized patient care, underscores the role of AI as a supportive tool rather than a replacement for human expertise. The integration of AI into primary care could thus serve as a critical step forward in the quest for improved clinical outcomes and adherence to established medical guidelines.

In the evolving landscape of otolaryngology, AI technologies like GPT-4 present transformative potential for research methodologies. Their capacity to distill complex data sets and rapidly evolving guidelines into actionable insights heralds a new era in personalized otologic care. However, this technological advancement does not come without challenges. The dependency of AI on existing datasets underscores the necessity for continual updates and ethical vigilance, particularly in data security and algorithmic biases. Future research should pivot toward integrating these AI tools into clinical practice, assessing their real-world impact on patient outcomes and decision-making processes. Such integration not only promises to augment the clinician’s expertise but also catalyzes a paradigm shift in how otologic research is conducted, analyzed, and applied.

## CONCLUSION

GPT-4 demonstrated high levels of accuracy and completeness when queried about otologic CPGs. Given the vast amount of medical literature and the rapid evolution of clinical knowledge, AI models like GPT-4 could play a significant role in assisting otolaryngologists and patients in accessing and interpreting medical information related to otologic clinical issues. Our study not only demonstrates GPT-4’s utility in otology but also sets a methodological foundation for future research in the integration of AI tools in otological studies and clinical practice. However, its application should be considered supplementary, augmenting the expertise of otologists and neurotologists rather than replacing their critical judgment. Future research should delve into how AI tools can be integrated more effectively into the diverse aspects of otologic care, particularly assessing their impact on patient outcomes and clinical decision-making.

## FUNDING SOURCES

None declared.

## CONFLICTS OF INTEREST STATEMENT

None declared.

## DATA AVAILABILITY STATEMENT

This data set is available upon reasonable request from the corresponding author.

## Supplementary Material


